# Multicenter performance evaluation of the Simplexa *C. auris* Direct assay for the detection of *Candida auris* colonization in bilateral axilla/groin swabs

**DOI:** 10.1128/jcm.01741-25

**Published:** 2026-06-29

**Authors:** Shadi Sepehri, Bradley Schindel, Suzane Silbert, Laura Rowe, Anna Marchese, Edward Willison, Omai Garner, Sukantha Chandrasekaran, Gregory J. Berry, Alamelu Chandrasekaran, Sarah Elliott, Brian Bernier, Janet Farhang, Kamal Kamboj, Preeti Pancholi

**Affiliations:** 1 BayCare Health System, Inc., Clearwater, Florida, USA; 2 Tampa General Hospital 7829 , Tampa, Florida, USA; 3 IRCCS Azienda Ospedaliera Metropolitana 845693 , Genoa, Italy; 4 Dipartimento di Scienze Chirurgiche e Diagnostiche Integrate (DISC), University of Genoa 9302 , Genoa, Italy; 5 Department of Pathology and Laboratory Medicine, University of California Los Angeles 8783 , Los Angeles, California, USA; 6 Center for Advanced Laboratory Medicine, Department of Pathology and Cell Biology, Columbia University Medical Center, New York, New York, USA; 7 Diasorin, Cypress, California, USA; 8 Luminex, Austin, Texas, USA; 9 Department of Pathology, The Ohio State University Wexner Medical Center, Columbus, Ohio, USA; University of Calgary, Calgary, Alberta, Canada

**Keywords:** *C. auris*, screening, colonization, PCR, culture

## Abstract

*Candida auris* is a multidrug-resistant fungal pathogen associated with healthcare outbreaks and high mortality. Its accurate and timely identification is critical for infection prevention, yet conventional culture-based methods are limited by slow growth, morphological ambiguity, and potential misidentification. This multicenter study evaluated the performance of the Simplexa *C. auris* Direct assay using 2,020 axilla/groin swab specimens collected from six clinical sites compared to culture followed by matrix-assisted laser desorption ionization-time of flight mass spectrometry (MALDI-TOF MS). Specimens were tested prospectively (*n* = 1,930) and retrospectively (*n* = 90), with demographic data spanning inpatient, intensive care unit, emergency department, and long-term acute care hospital settings. Discordant results were resolved by bi-directional sequencing, targeting the internal transcribed spacer and D1/D2 regions of the 28S ribosomal DNA gene. Compared to culture followed by MALDI-TOF MS identification, the Simplexa assay demonstrated an overall sensitivity of 94.8% and specificity of 98.7%, with a Cohen’s kappa of 0.80, indicating strong agreement. Discordance rates were low (1.4%), and diagnostic accuracy was high (98.6%). The limit of detection was 127 CFU/mL for Clade I and 260 CFU/mL for Clade IV. Mean cycle threshold values were significantly lower in culture-positive specimens (25.1, 95% CI, 23.6–26.7) compared to culture-negative ones (33.6, 95% CI, 32.1–35.0; *P* < 0.0001). Comparative analysis with four lab-developed molecular tests (LDTs) showed low rates of discordance: 0.9% for LDT 1 and 2, 1.5% for LDT 3, and 0% for LDT 4, indicating a high level of agreement between the molecular methods. The Simplexa *C. auris* Direct assay provides a rapid and reliable alternative to traditional methods, providing results in under 2 h, supporting early detection and containment of *C. auris* in healthcare settings.

**IMPORTANCE:**

Rapid and accurate detection of *Candida auris* colonization is essential for preventing healthcare-associated outbreaks and reducing mortality. This multicenter evaluation demonstrates that the Simplexa *C. auris* Direct assay offers a sensitive, specific, and practical alternative to culture-based methods, enabling earlier identification and containment of *C. auris*. These findings provide strong evidence to support its implementation in routine infection prevention strategies across diverse healthcare settings.

## INTRODUCTION

*Candida auris* has rapidly emerged as a major public health threat and an increasing source of healthcare-associated infections (HAIs), particularly among patients with underlying medical conditions (1, 2). This multidrug-resistant fungal pathogen has triggered numerous outbreaks worldwide, with substantial transmission in healthcare settings (3–7). Its rapid spread both within and across healthcare systems is driven by several compounding factors, including challenges in accurate identification, frequent resistance to available antifungal agents, and a unique capacity for persistent colonization of biotic and abiotic surfaces. These challenges are further exacerbated by the organism’s environmental hardiness and resistance to standard disinfectants (8–12).

The global rise of *C. auris* has prompted urgent attention due to its capacity to cause outbreaks in high-risk settings, including intensive care units and long-term care facilities. In the United States, more than 10,000 clinical cases have been reported since 2016, with over 4,500 cases documented in 2023 alone; surveillance data continue to demonstrate increasing case numbers and ongoing transmission (3, 4). Its expanding geographic distribution and ability to evade conventional detection and treatment strategies have positioned *C. auris* as a pathogen of significant concern, necessitating coordinated surveillance and response efforts worldwide (13, 14).

The whole-genome sequencing of clinical *C. auris* isolates has identified six genetically distinct clades, each exhibiting unique virulence factors, clinical features, and antifungal resistance profiles. These include clade I (South Asian), clade II (East Asian), clade III (African), clade IV (South American), and the more recently identified clade V (Iranian) and clade VI (Indomalayan/Bangladeshi) (12, 15). Clade I and clade III are geographically widespread, whereas clade II and clade IV have been associated with local epidemics (16). All four major clades have been detected in the United States, with clade I being the predominant and most concerning (17, 18). Notably, clades I and IV have been linked to higher rates of bloodstream infection and mortality (15, 16).

Although no definitive MIC breakpoints exist for antifungal susceptibility of *C. auris* isolates, the Centers for Disease Control and Prevention (CDC) has proposed tentative interpretive criteria based on the breakpoints of closely related *Candida* species and expert consensus (19). *C. auris* exhibits a very high level of resistance to fluconazole (>90%) and a moderate level of resistance to amphotericin B, ranging from 12% to 35%, leaving echinocandins as the best choice of treatment with only a 1%–12% resistance rate (10, 16, 18, 20). Among echinocandins, micafungin and anidulafungin demonstrate superior efficacy for *C. auris,* each associated with approximately 1% resistance, compared to caspofungin, which shows a resistance rate of up to 12% (16). Still, the risk of treatment failure and relapse of infection after antifungal therapy is higher with *C. auris* than with other *Candida* species (12).

Timely screening, accurate identification, and robust infection prevention measures are essential in preventing the spread of this opportunistic pathogen (1, 21). Among the diagnostic methods available for *C. auris* detection, molecular techniques have been proven to be rapid, high-throughput, and analytically sensitive, making them the preferred choice for routine screening (12, 22–26). Here, we studied the performance of the Simplexa *C. auris* Direct, the first FDA-authorized molecular assay for the detection of *C. auris* colonization from composite axilla/groin swabs.

## MATERIALS AND METHODS

### Study design

Six clinical laboratories, five across the United States and one in Italy, participated in this FDA clinical trial to evaluate the performance of the Simplexa *C. auris* Direct assay in composite bilateral axilla/groin swab specimens compared to the gold standard culture followed by matrix-assisted laser desorption ionization-time of flight mass spectrometry (MALDI-TOF MS).

Each study site obtained IRB approval or waiver based on its role in the study, and all residual samples (ESwab in Liquid Amies) were de-identified prior to enrollment in the study and testing. A total of 2,020 individual patient specimens were included in this study.

### Prospective specimens

A total of 1,930 prospective specimens (ESwab in Liquid Amies) were collected between April and July 2023. All specimens were first tested using the standard-of-care (SOC) method at each site (see Standard-of-care comparators for more details), followed by culture coupled with MALDI-TOF MS for confirmation and the Simplexa *C. auris* Direct assay. Residual, de-identified bilateral axilla/groin swabs from patients undergoing *C. auris* colonization screening at each study site were included if they met storage (15°C–25°C ≤ 48 h; 2°C–8°C ≤ 3 days; or ≤ −70°C thereafter), volume (≤340 µL), and transport-medium criteria (Liquid Amies ESwab; Copan 480C/480CE/490CE or BD 220245, or equivalent). Specimens failing to meet these criteria were excluded.

### Retrospective specimens

To increase the *C. auris* positivity rate, 90 retrospective specimens (ESwab in Liquid Amies) were also included in the study. These specimens had previously undergone SOC testing and were subsequently tested either fresh or frozen using the Simplexa *C. auris* Direct assay and plated for culture, followed by MALDI-TOF MS identification. All retrospective specimens met storage (15°C–25°C ≤ 48 h; 2°C–8°C ≤ 3 days; or ≤ −70°C thereafter) and transport medium (Liquid Amies ESwab; Copan 480C/480CE/490CE or BD 220245, or equivalent) testing criteria. Of the 90 retrospective specimens included in the study, only 11 (12.2%) were stored frozen after SOC testing. The average interval between specimen collection and study testing was 50 days (range, 12–98 days). All 11 frozen specimens tested positive by the Simplexa *C. auris* Direct assay, grew in culture, and were confirmed as *C. auris* by MALDI-ID, indicating that freezing did not adversely affect detection or recovery within the timeframes assessed in the present study.

### Clinical and demographic data

Clinical and demographic information was collected for all specimens, including patient age, sex, geographic location, clinical care setting, date/time of collection, storage conditions, and SOC method used.

### Simplexa *C. auris* Direct

The Simplexa *C. auris* Direct assay (Diasorin, Cypress, CA, USA) was performed on the LIAISON MDX system according to the manufacturer’s instructions. Briefly, 50 µL of reaction mix and 50 µL of patient specimen (ESwab in Liquid Amies) were sequentially loaded into the direct amplification disc after mixing the sample with the Sample Processing Solution. The disc was then placed on the instrument to initiate real-time PCR.

The assay targets a conserved region of the ITS2 gene for *C. auris* detection and includes an internal control to monitor PCR inhibition or failure. Data analysis, including amplification curves and Ct values, was performed using LIAISON MDX Studio Software. Total assay run time was approximately 105 min.

### Comparator methods

Simplexa *C. auris* Direct results were compared with culture followed by MALDI-TOF MS identification. Discordant results were resolved using a Diasorin-developed real-time PCR assay followed by bi-directional sequencing (BDS). A specimen was considered positive when culture with MALDI-TOF MS identification was *C. auris* positive and negative when both culture and MALDI-TOF MS identification were negative for *C. auris*. All specimens were tested using the SOC methods, culture/MALDI-TOF MS, and the Simplexa *C. auris* Direct assay at each study site, while discordant BDS testing was performed by Diasorin.

### Culture

Culture was inoculated from either fresh or frozen specimens. Specimens were stored at room temperature if tested within 48 h from collection, refrigerated (2°C–8°C) if tested within 3 days of collection, or frozen (≤ −70°C) if tested more than 3 days after specimen collection. Before culture was initiated, aliquots for molecular testing were prepared to prevent cross-contamination.

In brief, HardyCHROM Candida + auris plates (Hardy Diagnostics, Santa Maria, CA, USA) were inoculated and streaked for isolation, incubated at 35°C, and examined daily for up to 5 days. Samples were deemed negative if no growth was observed after 5 days. Colonies with the characteristic green “bullseye” morphology and UV fluorescence were presumptively identified as *C. auris*, subcultured onto Sabouraud Dextrose agar with antibiotics, incubated at 35°C–42°C up to 72 h, and then processed for MALDI-TOF MS confirmation.

### MALDI-TOF MS identification

*C. auris* identification was performed using FDA-cleared IVD libraries on either the bioMérieux VITEK MS (IVD v3.2) or Bruker MALDI Biotyper 2.0 Microflex LT (CA system library claim 4). Testing followed the manufacturer’s instructions. A VITEK MS confidence score of >99% or a Bruker log(score) >2.0 was required for species-level identification. All identifications were included in the study analysis.

### Bi-directional sequencing assay

A bi-directional sequencing assay was developed and validated by Diasorin for discordant analysis when the Simplexa *C. auris* Direct assay result was detected, and the combined culture followed by MALDI-TOF MS reference method result was not detected. The BDS assay targets the ITS and D1/D2 regions of the 28S rDNA gene for *C. auris* identification.

Discordant specimens (30 µL) were first treated using the Yeast Protein Kit (Zymo Research) and subsequently extracted on the easyMAG system (bioMérieux). Extracted material was stored at −80°C until amplification. PCR products were generated using primers from Integrated DNA Technologies and treated with Exonuclease I and Shrimp Alkaline Phosphatase to remove residual primers and nucleotides. Samples were then subjected to bi-directional sequencing using M13-labeled primers with BigDye chemistry on the ABI 3730xl DNA Analyzer. The resulting sequences were aligned and compared to GenBank using BLAST for species confirmation.

### Standard-of-care comparators

The SOC testing methods varied by study site. Four of the six study sites had *C. auris* PCR laboratory-developed tests (LDTs) as part of their site SOC testing, and two of the study sites used culture-based methods as their SOC test method. The methods for each LDT are outlined below.

#### LDT 1 and LDT 2

*C. auris* real-time PCR testing at Sites 5 and 6 was performed using the BioGX Sample-Ready *C. auris* rt-PCR reagents (BioGX, Inc., USA) on the BD MAX open testing system. The BioGX primers target the ITS2 region and include a sample processing control (SPC) compatible with the BD MAX DNA-3 extraction kit (BD, Inc., USA). For both LDTs, 200 µL of patient sample (ESwab in Liquid Amies) was transferred to a BD sample tube and loaded onto the instrument. Thermal cycling parameters were identical across sites, consisting of an initial hold at 99°C (300 s) followed by 40 cycles of 99°C (10 s), 58°C (29.5 s), and 70°C (14.5 s). Fluorescence detection was performed using the 585/630 channel for *C. auris* and the 680/715 channel for the SPC.

#### LDT 3

At Site 1, *C. auris* real-time PCR testing was performed on the cobas 6800 System using CDC-published ITS2 primers and a Roche-modified probe (Roche Diagnostics, Switzerland), prepared with the cobas omni Utility Channel Reagent Kit. Primers and probe were reconstituted to 100 µM, pooled, mixed with MMx-R2, and loaded into the reagent cassette per the manufacturer’s instructions. Patient samples (200 µL ESwab in Liquid Amies) were processed with an integrated DNA Internal Control during extraction. Cycling conditions included sequential holds at 55°C (120 s), 60°C (360 s), and 65°C (240 s), followed by 5 cycles of 95°C (5 s) and 55°C (30 s), and 45 cycles of 91°C (5 s) and 58°C (25 s). Fluorescence detection was used for both the *C. auris* target and internal control.

#### LDT 4

At Site 3, *C. auris* real-time PCR testing was performed on the LIAISON MDX instrument using Diasorin primers/probe that target the ITS2 region (Diasorin, Cypress). For lysis, 50 µL of the patient specimen (ESwab in Liquid Amies) and 5 µL of fungal lysis solution were incubated at 60°C for 30 min, briefly vortexed, and centrifuged. PCR master mix (10 µL per reaction) included TA Master Mix, *C. auris* primers, DNA Internal Control template and primers/probe, and molecular-grade water. For each sample, 8 µL master mix and 2 µL lysate were loaded into the 96-well disc. Cycling parameters consisted of a 97°C hold (600 s), followed by 40 cycles of 97°C (10 s) and 60°C (30 s). Fluorescence detection monitored FAM (target) and Q670 (internal control).

### Analytical sensitivity/limit of detection

The limit of detection (LoD) of the Simplexa *C. auris* Direct assay was evaluated using *C. auris* Clade I isolate AR382 (CDC AR-panel) and Clade IV isolate Z485 (Zeptometrix, Buffalo, NY, USA) fungal stocks quantified by standard CFU plating. Serial dilutions were prepared in a pooled negative axilla/groin swab matrix. Seven dilutions were tested in six replicates to establish preliminary LoDs, followed by 60 replicates at the lowest concentration achieving 100% detection. Tested concentrations were as follows: for Clade I, 233, 193, 127, 113, 47, 20, and 10 CFU/mL; for Clade IV, 533, 303, 260, 190, 143, 80, and 53 CFU/mL. Final LoD was defined as the lowest concentration yielding ≥95% detection for each clade tested and was determined using percent detection and Probit analysis.

### Statistical analysis

Positive percent agreement (PPA), negative percent agreement (NPA), Probit, Cohen’s kappa, discordance rates, diagnostic accuracy, positive and negative likelihood ratios, and two-sided (upper/lower) 95% confidence intervals (CIs) were calculated using Microsoft Office Excel 365 MSO software (Microsoft, Redmond, WA, USA) and Prism GraphPad software (GraphPad Software Inc., San Diego, CA, USA). The positive percent agreement was calculated as TP/(TP + FN) × 100, and the negative percent agreement was calculated as TN/(TN + FP) × 100, where TP is true-positive results, FN is false-negative results, TN is true-negative results, and FP is false-positive results. Probit analyses were used for the CFU/mL determination of the analytical sensitivity study (27). Diagnostic accuracy was calculated as [(TP + TN)/(TN + FP + FN +TP)]. Cohen’s kappa values (*κ*) were calculated as a measure of the overall agreement, categorized as almost perfect (>0.90), strong (0.80–0.90), moderate (0.60–0.79), weak (0.40–0.59), minimal (0.21–0.39), or none (0–0.20). The discordance rate was calculated as [(FP + FN)/total number of samples tested] × 100. The positive likelihood ratio (LR+) was calculated as sensitivity/(1 − specificity), and the negative likelihood ratio (LR−) was calculated as (1 − sensitivity)/specificity. A likelihood ratio >1 indicates that the test result is associated with the presence of the disease, whereas a likelihood ratio <1 indicates that the test result is associated with the absence of the disease. Positive likelihood ratios that are >10 are considered strong evidence for the presence of the disease, whereas negative likelihood ratios <0.1 are considered strong evidence for the absence of the disease (28).

## RESULTS

### Clinical specimen demographics

**Fig 1:**
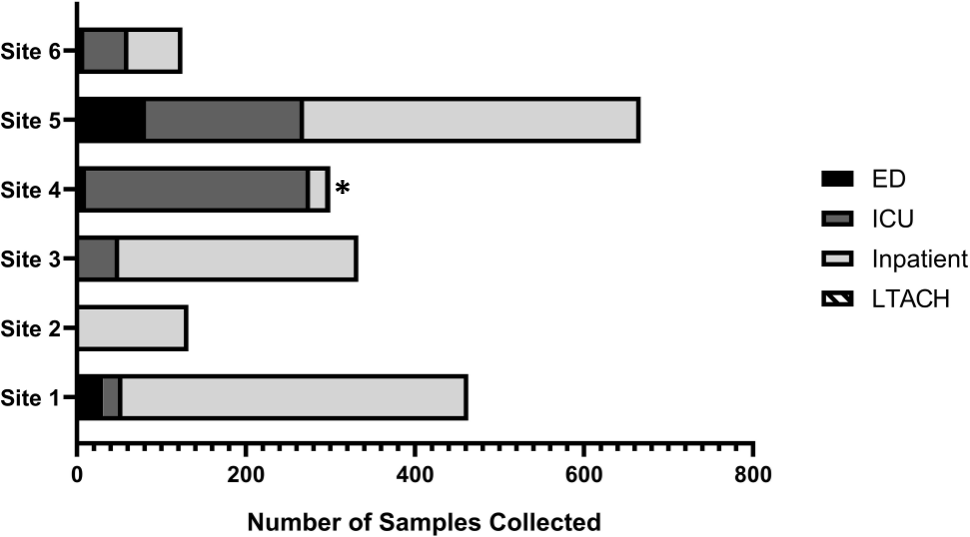
Breakdown of the number of clinical specimens collected by each study site. A total of 2,020 specimens were collected from all sites. The specimens are broken down into categories related to the location of the patient when the specimen was collected: ED, ICU, inpatient (before admission), and LTACH. Only one specimen was collected from an LTACH location, and it is denoted by an asterisk (*) on the graph.

A total of 2,020 composite bilateral axilla/groin swab specimens were collected across six clinical study sites from patients undergoing *C. auris* colonization screening (Fig. 1). Specimens were obtained from a range of care settings, including inpatient units, intensive care units (ICUs), emergency departments (EDs), and long-term acute-care hospitals (LTACHs). Although most specimens originated from inpatients, the distribution of collection locations varied among sites, reflecting differences in local *C. auris* screening practices.

Patient demographic characteristics are summarized in Table 1. Most specimens were collected from adults aged 18–65 years (49.4%) and adults >65 years (49.1%), with only 1.4% from patients younger than 18 years. Specimen collection occurred primarily in inpatient settings (64.8%), followed by ICUs (28.6%), EDs (6.6%), and LTACHs (<0.1%). The study population was balanced by sex, with 51.6% male and 48.4% female patients.

**TABLE 1:** Demographics of the specimens included in this study^*a*^

Characteristic	No. (%)
Patient age (yrs)	
0–18	28 (1.4%)
>18–65	998 (49.4%)
>65	992 (49.1%)
Unknown	2 (0.1%)
Total	2,020 (100%)
Patient location	
ED	133 (6.6%)
ICU	577 (28.6%)
Inpatient	1,309 (64.8%)
LTACH	1 (<0.1%)
Total	2,020 (100%)
Patient gender	
Male	1,042 (51.6%)
Female	978 (48.4)
Total	2,020 (100%)

^*a*^ A total of 2,020 specimens were collected and included in this study. The specimens are broken down into different demographic categories, including patient age, the location of the patient when the specimen was collected, and gender. ED, emergency department; ICU, intensive care unit; inpatient (before admission); LTACH, long-term acute care hospital.

### Simplexa *C. auris* Direct clinical performance

A total of 1,930 prospective and 90 retrospective clinical specimens were included in the analysis. The clinical agreement of the Simplexa *C. auris* Direct assay compared to the reference method, culture followed by MALDI-TOF MS identification, is outlined in Table 2. The sensitivity of the prospective specimens compared to the reference method was 94.1% (95% CI, 0.81–0.98), and the specificity was 98.8% (95% CI, 0.98–0.99), with a Cohen’s kappa value of 0.72, representing moderate agreement. The sensitivity of the retrospective specimens compared to the reference method was 95.8% (95% CI, 0.80–0.99), and the specificity was 95.5% (95% CI, 0.87–0.98), with a Cohen’s kappa value of 0.89, representing strong agreement. Finally, taken together, the overall combined sensitivity was 94.8% (95% CI, 0.96–0.98) and specificity was 98.7% (95% CI, 0.98–0.99), with a Cohen’s kappa value of 0.80, representing strong agreement.

**TABLE 2:** Simplexa *C. auris* Direct performance compared to the reference method

Simplexa *C. auris* Direct	Reference method Culture + MALDI ID			±95% CI			Discordance rate (%)	Diagnostic accuracy (%)	Likelihood ratios^*a*^
	Positive	Negative	Total	Sensitivity	Specificity	Kappa (*κ*)			
Prospective									
Positive	32	22^*c*^	54	94.1% (0.81–0.98)	98.8% (0.98–0.99)	0.72 (0.61–0.83)	1.2	98.8	LR+ 58.81 LR− 0.059
Negative	2^*b*^	1,874	1,876						
Total	34	1,896	1,930						
Retrospective									
Positive	23	3^*e*^	26	95.8% (0.80–0.99)	95.5% (0.87–0.98)	0.89 (0.78–1)	4.4	95.6	LR+ 21.29 LR− 0.043
Negative	1^*d*^	63	64						
Total	24	66	90						
Combined									
Positive	55	25	80	94.8% (0.86–0.98)	98.7% (0.98–0.99)	0.8 (0.71–0.87)	1.4	98.6	LR+ 72.92 LR− 0.052
Negative	3	1,937	1,940						
Total	58	1,962	2,020						

^*a*^ LR+, likelihood ratio of a positive result; LR−, likelihood ratio of a negative result.

^*b*^ The two false-negative specimens also tested negative by BDS. One of the false-negative specimens had an available SOC PCR result, which was also negative.

^*c*^ Of the 22 false-positive specimens, 21 were tested by BDS, 7 tested positive and 14 tested negative. Of the 14 false-positive specimens with available SOC PCR LDT results available, 7 tested positive and 7 tested negative.

^*d*^ The one false-negative specimen also tested negative by BDS. The available SOC LDT PCR result was positive.

^*e*^ Of the false-positive specimens tested by BDS, two tested positive and one tested negative. The three false-positive specimens also tested positive by the SOC PCR LDT.

There were 25 false-positive and 3 false-negative Simplexa *C. auris* Direct assay results in this study when compared to the culture-based reference method. These discordant specimens were further analyzed using a bi-directional sequencing assay. Of the 25 false-positive specimens, 24 were tested by BDS for discordant analysis (1 specimen had insufficient volume to allow BDS testing), and 9 resulted as positive, while 15 resulted as negative. Seventeen of the false-positive specimens also had standard-of-care PCR LDT results available, and of these, 10 were positive, and 7 were negative. There were three false-negative Simplexa *C. auris* Direct assay results, and all three specimens also tested negative by the BDS discordant analysis testing. Of the two false-negative specimens with available SOC PCR LDT results, one had a negative result, and one had a positive result.

Additional statistical analyses were performed to further evaluate agreement between the Simplexa *C. auris* Direct assay and the reference culture/MALDI-TOF MS method. Across prospective, retrospective, and combined specimen cohorts, discordance rates were low, 1.2%, 4.4%, and 1.4%, respectively, indicating a high level of agreement between methods (Table 2).

Consistent with these findings, diagnostic accuracy was high in all cohorts, measuring 98.8% for prospective specimens, 95.6% for retrospective specimens, and 98.6% overall (Table 2). The high diagnostic accuracy rates (>95%) suggest that the Simplexa *C. auris* Direct assay is an accurate method for the detection of *C. auris*.

To account for the relatively low *C. auris* prevalence in the study population, likelihood ratios were also calculated. Positive likelihood ratios were 58.51, 21.29, and 72.92 for the prospective, retrospective, and combined data sets, respectively, while negative likelihood ratios were 0.059, 0.042, and 0.052. Positive likelihood ratios >10 and negative likelihood ratios <0.1 across all analyses demonstrate that Simplexa *C. auris* Direct results are strongly associated with the true presence or absence of *C. auris* in patient specimens.

### Distribution of Ct values from the prospective specimens

**Fig 2:**
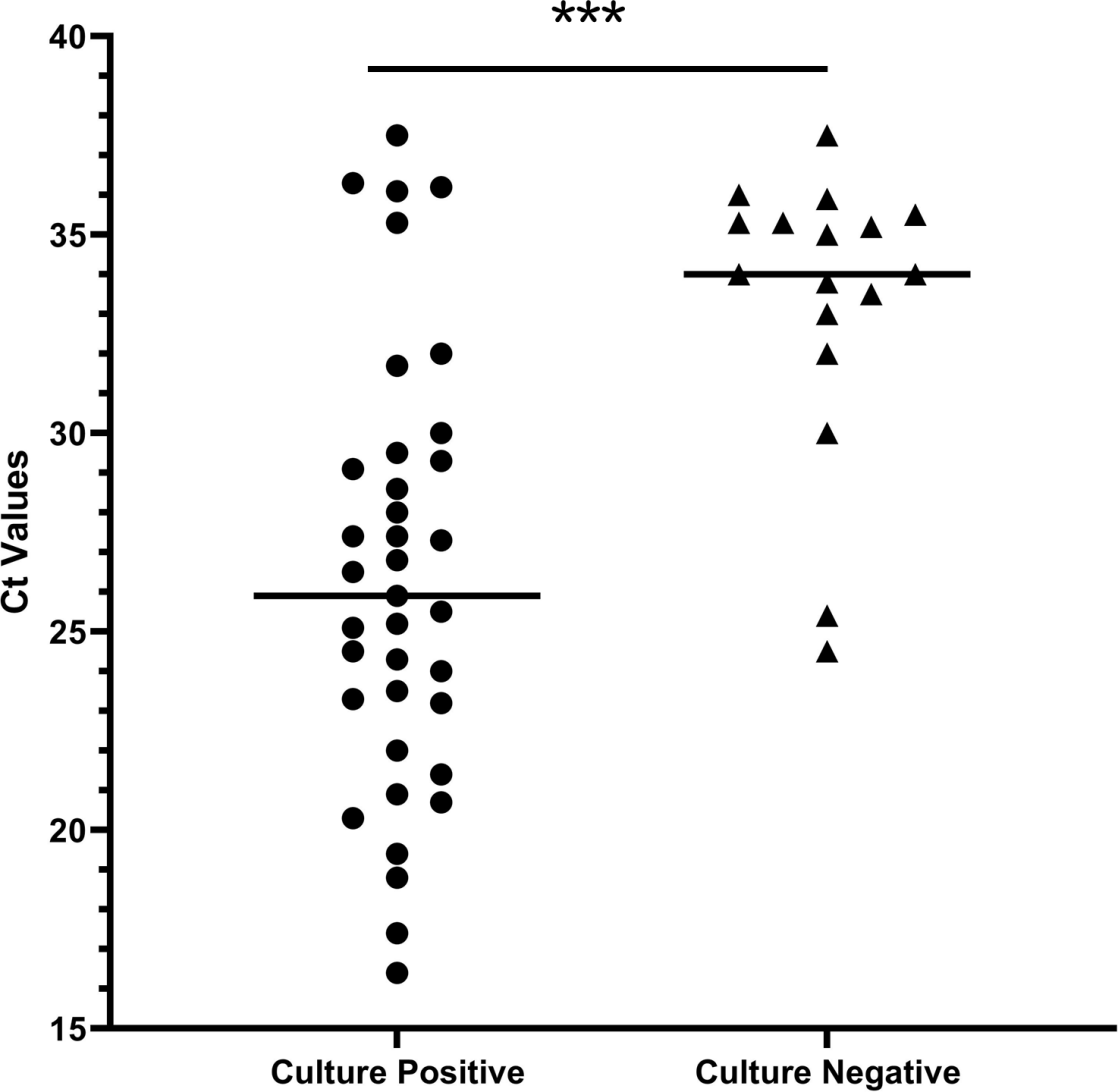
Distribution of Ct values from the prospectively collected specimen cohort. Of the 54 specimens detected by the Simplexa *C. auris* Direct Assay, 32 were culture positive, and 22 were culture negative. The mean Ct value for the culture-positive specimens was 25.13 (95% CI, 23.6–26.7) and 33.56 (95% CI, 32.1–35.0) for culture-negative specimens. The difference between the mean Ct values was statistically significant (***P-value < 0.0001).

There were 54 positive prospective clinical specimens detected by the Simplexa *C. auris* Direct assay in this study. Of those positive specimens, 32 were culture positive with a *C. auris* identification confirmed by MALDI-TOF MS, 17 were culture negative, and 5 were culture positive with a non-*C*. *auris* yeast identification by MALDI-TOF MS (Table 3). Of these five specimens, four had SOC LDT PCR results available: one was *C. auris* positive, and three were negative. The mean Ct value of the latter five specimens was 34.5, ranging between 27.4 and 37.5, suggesting that the burden of *C. auris* in the specimens was possibly too low to be detected by culture. These five specimens were considered *C. auris* culture negative. The difference between the mean Ct values of the 32 culture-positive and 22 culture-negative specimens, 25.13 (95% CI, 23.6–26.7) and 33.56 (95% CI, 32.1–35.0), respectively, was statistically significant (*P*-value < 0.0001) (Fig. 2). The mean Ct values from positive specimens were also assessed by patient age group and sex, and no statistically significant differences were observed (*P*-value > 0.05; data not shown).

**TABLE 3:** Reduced culture recovery of *C. auris* at high Ct values^*a*^

Specimen no.	Simplexa *C. auris* Direct Ct	MALDI-TOF identification
1	35.3	*Candida tropicalis*
2	27.4	*Candida parapsilosis*
3	36.1	*Candida krusei*, *Candida albicans*, *C. parapsilosis*
4	36.2	*Candida lusitaniae*
5	37.5	*C. albicans*, *C. tropicalis*

^*a*^ Five prospective specimens that were detected by the Simplexa *C. auris* Direct assay had only non-*C. auris* organisms identified by culture/MALDI-TOF. The mean Ct value of *C. auris* in these specimens was 34.5.

### Analytical sensitivity

The LoD, defined as the lowest concentration detected in ≥95% of replicates by percent positive rate, was evaluated using two approaches: manufacturer-stated concentrations and plate-based CFU enumeration. Both methods used the same dilution series of *C. auris* Clade I and Clade IV isolates. Although all four major clades circulate in the United States and Clades I, III, and IV predominate, Clades I and IV were selected because they are most frequently associated with invasive bloodstream infections (15–18).

Using manufacturer-provided concentrations, the LoD was 300 CFU/mL for Clade I and 400 CFU/mL for Clade IV (Table 4). Both clades showed a 98.3% detection rate (59/60). Mean Ct values at the LoD were similar, 28.4 ± 1.4 (4.9% CV) for Clade I and 27.9 ± 1.3 (4.6% CV) for Clade IV, indicating comparable assay performance. This corresponds to 3 CFU/reaction for Clade I and 4 CFU/reaction for Clade IV. Probit analysis yielded 95% detection thresholds of 267 ± 13 CFU/mL and 284 ± 15 CFU/mL for Clades I and IV, respectively.

**TABLE 4:** Summary of limit of detection

*C. auris*	Positive rate %						Probit (±95% CI)			
	Commercial control		CFU plating		% Detection	Mean Ct ± SD (%CV)	Commercial control		CFU plating	
	CFU/mL	CFU/Rxn^*a*^	CFU/mL	CFU/Rxn			CFU/mL	CFU/Rxn	CFU/mL	CFU/Rxn
Clade I	300	3	127	2	98.3% (59/60)	28.4 ± 1.4 (4.9%)	267 ± 13	3	76 ± 13	1
Clade IV	400	4	260	3	98.3% (59/60)	27.9 ± 1.3 (4.6%)	284 ± 15	3	196 ± 15	2

^*a*^ Rxn, reaction.

Using CFU values determined by plate counting, the LoD was 127 CFU/mL for Clade I and 260 CFU/mL for Clade IV. Probit analysis refined these estimates to 76 CFU/mL for Clade I and 196 CFU/mL for Clade IV (Table 4).

### Simplexa *C. auris* IVD results compared to the site standard-of-care molecular LDT results

A summary of the Simplexa *C. auris* Direct assay performance compared with SOC PCR LDTs across four study sites is shown in Table 5. Because of their similar methods, the LDT 1 and LDT 2 results were combined. Performance varied by site, likely reflecting differences in LDT design and sample numbers.

**TABLE 5:** Simplexa *C. auris* IVD results compared to the site standard-of-care molecular LDT results

Molecular comparator	Simplexa *C. auris* Direct			±95% CI			Discordance rate (%)	Diagnostic accuracy (%)
	Positive	Negative	Total	PPA^*a*^	NPA^*b*^	Kappa (*κ*)		
LDT 1 and 2								
Positive	14	2^*c*^	14	73.7% (0.51–0.88)	99.7% (0.99–1)	0.80 (0.64–0.95)	0.9	99.1
Negative	5^*d*^	771	771					
Total	19	773	792					
LDT 3								
Positive	22	4^*e*^	26	88.0% (0.69–0.97)	99.1% (0.98–0.99)	0.85 (0.75–0.96)	1.5	98.5
Negative	3^*f*^	434	437					
Total	25	438	463					
LDT 4								
Positive	1	0	1	100% (0.17–1)	100% (0.99–1)	1 (1–1)	0	100
Negative	0	332	332					
Total	1	332	333					

^*a*^ PPA, positive percent agreement.

^*b*^ NPA, negative percent agreement.

^*c*^ One false-positive sample was positive by culture but negative by MALDI ID, and one false-positive sample was negative by culture.

^*d*^ Of the five false-negative samples, one was positive by culture and MALDI ID, one was positive by culture but negative by MALDI ID and BDS, two were negative by culture and BDS, and one was negative by culture but positive by BDS.

^*e*^ Of the four false-positive samples, three were negative by culture and one was positive by culture and MALDI ID but negative by BDS.

^*f*^ The three false-negative samples were negative by culture and BDS.

The PPA was 73.7% (95% CI, 0.51–0.88) for combined LDT 1 and 2, 88% (95% CI, 0.69–0.97) for LDT 3, and 100% (95% CI, 0.17–1.00) for LDT 4. The NPA was 99.7% (95% CI, 0.99–1.00), 99.1% (95% CI, 0.98–0.99), and 100% (95% CI, 0.99–1.00) for LDT 1 and 2, LDT 3, and LDT 4, respectively.

Cohen’s kappa values indicated strong agreement with the Simplexa assay for LDT 1 and 2 (0.80; 95% CI, 0.64–0.95) and LDT 3 (0.85; 95% CI, 0.75–0.96) and perfect agreement for LDT 4 (1.0; 95% CI, 1–1).

Discordance rates were low between all of the SOC LDTs and the Simplexa *C. auris* Direct assay, indicating high rates of agreement: 0.9% for LDT 1 and 2, 1.5% for LDT 3, and 0% for LDT 4. Diagnostic accuracy was 99.1%, 98.5%, and 100% for LDT 1 and 2, LDT 3, and LDT 4, respectively.

### Additional non-*C*. *auris* MALDI-TOF MS organism identifications

There are many organisms capable of growing on the chromogenic agar used in this study, and each distinct colony type was processed for MALDI-TOF MS identification. The non-*C*. *auris* organisms present in cultures in this study are outlined in Fig. S1. As expected, the most commonly identified species were *Candida albicans* (36.98%), *Candida glabrata* (25.28%), *Candida parapsilosis* (14.72%), and *Candida tropicalis* (8.30%). Several other *Candida* species also grew on the chromogenic agar, though each organism was present in less than 4% of the identifications. While most of the non-*C*. *auris* organisms identified were other *Candida* species, there were some non-*Candida* organisms identified as well, including *Malassezia*, *Cryptococcus*, *Lodderomyces*, *Rhodotorula*, and *Trichosporon* species (Fig. S1).

## DISCUSSION

Despite its clinical significance, the laboratory detection and identification of *C. auris* remains challenging. *C. auris* isolates can be recovered from a variety of body sites—both sterile and nonsterile—including skin, nares, ear, urine, wound, and respiratory specimens (29). However, the morphological features of *C. auris* are indistinguishable from those of other *Candida* species when using standard culture media, complicating its accurate identification. Historically, species-level identification of *Candida* isolates from nonsterile sites has not been routinely performed. In contrast, CDC recommends species-level identification in specific scenarios, such as when a confirmed case of *C. auris* has been detected within a healthcare facility or when a patient has had a healthcare exposure in a region where *C. auris* has been reported. These recommendations may disrupt established workflows in clinical microbiology laboratories (29). Moreover, accurate identification of *C. auris* proved difficult, especially when relying on morphologic, biochemical, and phenotypic characteristics of isolates (30). Misidentifications of *C. auris* by commercial diagnostic platforms as other closely related species of *Candida*, such as *Candida haemulonii*, *Candida duobushaemulonii*, *Candida faemata* (*Debaryomyces hansenii*), or *Candida lusitaniae* (*Clavispora lusitaniae*), have been widely reported and were likely due to lack of representation in reference databases (12, 19, 23, 29–31); however, subsequent library enrichment in several commercial systems has markedly improved the accuracy of *C. auris* identification.

Timely identification of patients colonized with *C. auris* is essential for preventing transmission, as early detection enables rapid implementation of infection control measures and limits environmental contamination from colonized patients who readily shed the organism (32, 33). Colonization with *C. auris* may also progress to invasive clinical infections, which carry crude mortality rates ranging from 30% to 72% (34). A recent study reported that 6%–7% of colonized patients in the United States developed clinical infections between 2016 and 2023 (35). Implementing effective screening protocols, ideally upon admission, enables healthcare facilities to adopt a proactive strategy for managing *C. auris* (36), thereby reducing the incidence of invasive infections through preventive measures rather than relying solely on treatment.

Culture-dependent screening methods, while widely used, share some inherent limitations due to the nature of the process. First, the time required for organism growth and subsequent identification can span several days, which is suboptimal in the context of a HAI that necessitates prompt isolation and infection control interventions. Second, *C. auris* is morphologically indistinguishable from other *Candida* species and skin-colonizing yeasts, and its growth can be easily obscured by overgrowth of other organisms on standard culture media (21). In our study, axilla/groin swabs frequently yielded a diverse array of skin colonizers, with *C. albicans*, *C. glabrata*, *C. parapsilosis*, and *C. tropicalis* being the most commonly recovered species (Fig. S1). Although chromogenic media can aid in differentiating *C. auris* from other major *Candida* species, the development of distinctive colony color and hue typically requires up to 48 h incubation and lacks sufficient specificity (21). Third, despite being considered the gold standard, culture is not analytically as sensitive as molecular methods. Korsten et al. (21) showed that the sensitivity of culture for *C. auris* decreases at lower concentrations and in the presence of other species. This is in alignment with the present study, where we had five specimens that were positive by the Simplexa *C. auris* Direct assay with relatively high Ct values, but negative for *C. auris* by culture and MALDI-TOF MS. Remarkably, all five samples had one or more other *Candida* species identified by MALDI-TOF MS (Table 3). It is possible that the combination of the low burden of *C. auris* in the specimen and the presence of other *Candida* species contributed to the decreased sensitivity of culture compared to the Simplexa *C. auris* Direct assay. Korsten et al. (21) suggested incubation at higher temperatures (42°C) to increase culture specificity. However, the growth of *C. auris* at 42°C is slow and cannot be relied on to differentiate *C. auris* from closely related yeasts like *C. haemulonii* (23). Similarly, enrichment of Sabouraud or yeast nitrogen-based broth with sugars as the carbon source and 10% NaCl, together with incubation at 42°C, enhanced the specificity of *C. auris* isolation from closely related species; however, *C. glabrata* and *C. parapsilosis* retained the ability to grow under these conditions (11). Other morphologic characteristics of *C. auris*, such as lack of pseudohyphae, are also non-specific and are exhibited by other *Candida* species such as *C. glabrata* (23).

Molecular identification of *C. auris* has been investigated by multiple research groups over the past decade and is increasingly regarded by some as the gold standard for screening (23–25, 37). In this study, the performance of Simplexa *C. auris* Direct was compared to culture followed by MALDI-TOF MS identification as the gold standard reference method, and it showed an overall sensitivity of 94.8% and specificity of 98.7% (Table 2). These findings demonstrate the high diagnostic accuracy of the Simplexa *C. auris* Direct assay and underscore its utility as a fast and reliable alternative to conventional methods, particularly in settings where timely detection is critical for infection prevention and control measures to limit the spread of this pathogen.

Among the 2,020 specimens tested using the Simplexa *C. auris* Direct assay, a small number showed discrepancies when compared to culture-based results. These discrepancies raise important questions about the comparative sensitivity of molecular versus culture-based diagnostics. Potential factors contributing to the three false‐negative Simplexa results include specimens with very low levels of *C. auris*, sampling variability that results in insufficient target DNA in the aliquot tested, or the presence of inhibitory or degraded material that reduces amplification efficiency due to suboptimal transport and storage conditions (including repeated freeze–thaw cycles). Follow-up testing on the 25 potentially false-positive cases yielded mixed outcomes: some cases were confirmed positive by BDS and SOC LDT PCR, although not all samples were available for both methods. False-positive results can lead to unnecessary isolation, repeat testing, and added patient anxiety, while also driving increased hospital costs through enhanced cleaning, contact precautions, and infection-prevention investigations. They may also inflate facility-level *C. auris* prevalence metrics, affecting surveillance data and resource allocation. Understanding and minimizing false positives is therefore important for both patient care and operational efficiency.

Further analysis revealed a significant difference in the mean Ct values between culture-positive and culture-negative specimens (Fig. 2), suggesting that molecular diagnostics may detect lower levels of *C. auris* than traditional culture methods. Whether this increased sensitivity translates to clinical relevance remains uncertain, as patient-level data were not assessed in this study.

We observed excellent diagnostic accuracy when comparing the performance of the Simplexa *C. auris* Direct assay with four laboratory-developed molecular methods, with agreement ranging from 98.5% to 100% (Table 5). We also found stronger concordance between culture and the Simplexa *C. auris* Direct assay than between culture and any of the four LDTs evaluated in this study (Table 5). These findings highlight the robust performance of Simplexa *C. auris* Direct, particularly in its agreement with culture-based methods. However, the variability in discordant resolution across assays underscores the need for further investigation into assay-specific sensitivity and the clinical implications of low-level detection. Additional data, including patient context and longitudinal outcomes, would be valuable in interpreting the significance of these molecular findings.

The clinical significance of *C. auris* is undeniable, given its rapid global spread, its resistance to multiple classes of antifungals, and its capacity to cause invasive infections associated with high mortality rates, which highlights the urgent need for rapid and reliable diagnostic methods to identify colonized patients and implement timely infection control measures. Screening for *C. auris* using culture-based methodologies—relying on morphological, biochemical, and phenotypic characteristics of isolates—remains challenging due to the inherently slow growth of the organism and the limitations of chromogenic media and commercial identification systems in accurately differentiating *C. auris* from closely related species. In contrast, the Simplexa *C. auris* Direct assay offers a rapid, accurate, and easy-to-use molecular option, representing a promising advancement in the global effort to combat this emerging pathogen.

## SUPPLEMENTAL MATERIAL

Figure S1 jcm.01741-25-s0001.tif A chart illustrating the breakdown of additional organisms identified by MALDI-TOF in the study.

ASM does not own the copyrights to Supplemental Material that may be linked to, or accessed through, an article. The authors have granted ASM a non-exclusive, world-wide license to publish the Supplemental Material files. Please contact the corresponding author directly for reuse.
